# Exploring the molecular mechanisms of phthalates in the comorbidity of preeclampsia and depression by integrating multiple datasets

**DOI:** 10.3389/fpsyt.2025.1596995

**Published:** 2025-12-02

**Authors:** Xinpeng Tian, Xia Gu, Yuecheng Yuan, Yaqiong Zhang, Yantuanjin Ma, Yingliang Liu

**Affiliations:** 1 Intensive Care Unit, the Xichang People’s Hospital, Xichang, Sichuan, China; 2 Kunming Medical University, Kunming, China; 3 Institute of Biomedical Engineering, Kunming Medical University, Kunming, China; 4 Department of Orthopaedics, the People’s Hospital of Chuxiong Yi Autonomous Prefecture, Chuxiong, Yunnan, China; 5 Department of Orthopaedics, The Second Affiliated Hospital of Kunming Medical University, Kunming, Yunnan, China

**Keywords:** plasticizers, preeclampsia, depression, immune, molecular docking

## Abstract

**Introduction:**

Preeclampsia (PE) and depressive disorder (DD) exhibit clinical comorbidity, yet the molecular mechanisms underlying this association remain poorly understood.

**Methods:**

Differential expression analysis of placental and peripheral blood transcriptomes was performed to identify PE-associated secretory protein genes. A depression-related coexpression network was constructed to obtain DD-related genes. Protein–protein interaction integration and functional enrichment analyses were then applied to identify shared regulatory pathways. Machine learning algorithms were applied to select core diagnostic genes, followed by validation in independent cohorts. A nomogram model was developed, and gene set enrichment, immune cell infiltration analysis, transcription factor regulatory mapping, and molecular docking with plasticizer compounds were conducted.

**Results:**

A total of 434 secretory protein genes were associated with PE, whereas the depression-related network identified 1,165 DD-associated genes. Immune-related pathways and extracellular-matrix remodeling emerged as common mechanisms. CLEC3B, CTLA4, and PDPR were identified as core diagnostic genes and showed robust predictive performance in the nomogram model. These genes were enriched in immune-related signaling pathways, including the B-cell receptor and NOD-like receptor pathways. Aberrant proportions of naïve CD4⁺ T cells were observed, and gene expression correlated with specific immune-cell populations. Multiple transcription factors were predicted to regulate the three genes. Molecular docking indicated stable interactions between the encoded proteins and plasticizer compounds, suggesting potential environmental contributions to comorbidity.

**Discussion:**

The findings provide molecular evidence linking vascular dysfunction in PE with immune-related mechanisms in DD and highlight potential biomarkers for early diagnosis and therapeutic intervention.

## 1 Introduction

PE is a severe pregnancy complication characterized by hypertension and multisystem dysfunction, usually occurring in the middle to late stages of pregnancy. It is a major contributor to maternal and perinatal morbidity and mortality worldwide (1). The global prevalence of PE ranges from 2% to 8%, with notable regional and population-specific differences (2). While its exact etiology remains elusive, its core mechanisms include abnormal placental development, oxidative stress, immune dysregulation, and endothelial dysfunction (3, 4). Depression, a common psychiatric disorder with a global prevalence of 8% (5), is particularly common among women, with significantly increased rates during the perinatal period (6).

In recent years, the comorbidity of PE and depression has gained increasing research focus (7). Evidence suggests that the pathophysiological changes associated with PE may increase the risk of perinatal and postpartum depression through mechanisms involving inflammatory cytokines, neuroendocrine regulation, and oxidative stress (8, 9). Conversely, depression may exacerbate the severity of PE by modulating immune function or placental activity (10, 11). However, the molecular basis of this comorbid relationship remains unclear, hindering the development of effective therapeutic and early intervention strategies. PE comorbid with depressive symptoms may have dual detrimental effects on maternal and neonatal outcomes, including preterm birth, fetal intrauterine growth restriction, delayed postpartum recovery, and impaired maternal–infant bonding (12, 13). Therefore, elucidating the interactive mechanisms between these conditions, particularly their intersecting molecular and cellular pathways, is critical for enhancing perinatal care quality and reducing adverse pregnancy outcomes.

Phthalates, including bisphenol A (BPA), dibutyl phthalate (DBP), and di(2-ethylhexyl) phthalate (DEHP), are widely utilized as plasticizers to increase the flexibility and extensibility of plastic products. These compounds are commonly found in food packaging materials, medical devices, and personal care products (14–16). Owing to their high lipophilicity and environmental persistence, phthalates tend to bioaccumulate within ecosystems and enter the human body via inhalation, ingestion, or dermal exposure, subsequently accumulating in adipose tissue, placenta, and other organs (17–19). Research increasingly suggests that plasticizer exposure disrupts endocrine function, impairs reproductive health, and is associated with metabolic and psychiatric disorders such as depression (20, 21). Recently, their role in PE has attracted attention, with studies linking maternal exposure to increased risk (22), although the mechanisms involved remain unclear. Potential pathways include oxidative stress induction, inflammatory activation, and placental dysfunction, which contribute to PE and its comorbidities. Notably, phthalates may exert critical effects within the neuroimmune-endocrine regulatory network by modulating hypothalamic–pituitary–adrenal (HPA) axis activity, regulating inflammatory cytokine expression, and disrupting neurotransmitter metabolism (23, 24). These combined actions may contribute to the pathogenesis of both preeclampsia (PE) and depression. Therefore, phthalate exposure represents a potential environmental risk factor for the comorbidity of PE and depressive disorders, warranting further mechanistic investigation.

Advances in bioinformatics and multiomics analyses have facilitated the investigation of the molecular mechanisms underlying disease comorbidities. Public gene expression databases, such as the Gene Expression Omnibus (GEO), offer valuable resources for decoding transcriptomic patterns in various diseases (25). Methods such as WGCNA, differential expression analysis, and functional enrichment analysis are instrumental in elucidating complex pathologies (26, 27). This study aims to integrate multiple datasets to investigate the potential molecular mechanisms underlying the comorbidity of PE and depression associated with plasticizer exposure. By analysing transcriptomic data from blood samples, we identified differentially expressed genes and coexpression modules linked to the comorbidity of PE and depression. Additionally, molecular docking was employed to investigate key genes and their functions associated with plasticizer exposure. Further research into plasticizer exposure and its potential targets may provide new theoretical foundations and intervention strategies for the early diagnosis and precision treatment of PE.

## 2 Materials and methods

### 2.1 Data acquisition and processing

Four transcriptomic datasets—GSE48424, GSE75010, GSE39653, and GSE98793—were obtained from the GEO database (https://www.ncbi.nlm.nih.gov/geo/). The GSE75010 dataset, comprising placental transcriptome data related to preeclampsia (PE), includes 77 control samples and 80 placental tissue samples from PE patients. The peripheral blood transcriptome dataset GSE48424 includes 18 control samples and 18 samples from PE patients. Additionally, GSE39653, containing PBMC transcriptomic data from patients with DD, was employed as the training set, consisting of 24 control samples and 29 samples from DD patients. The GSE98793 dataset, derived from microarray data of peripheral blood from DD patients, was used for validation. To ensure data comparability, gene expression matrices across all datasets were normalized via the “normalizeBetweenArrays” function in R. Differentially expressed genes (DEGs) were subsequently identified via the *limma* package, with selection thresholds set at FDR < 0.05 and |logFC| > 0.233. The resulting DEGs were visualized via the *pheatmap* and *ggplot2* packages to generate heatmaps and volcano plots that display the gene expression characteristics. A total of 3,946 human genes encoding secreted proteins were retrieved from the Human Protein Atlas database (https://www.proteinatlas.org/).

### 2.2 WGCNA for constructing coexpression networks

To identify gene modules strongly associated with the phenotype and to uncover hub genes within the network, weighted gene coexpression network analysis (WGCNA) was conducted. On the basis of the peripheral blood transcriptomic dataset GSE39653 from individuals with DD, a gene coexpression network was constructed via the “WGCNA” package in R. The 25% most highly variable genes among the differentially expressed genes were selected as inputs for network construction. A soft-thresholding power of β = 4 and a scale-free topology fit index of R² = 0.9 were applied to ensure that the resulting network conformed to a scale-free topology. Hierarchical clustering was then performed via the topological overlap matrix (TOM) to generate a gene dendrogram, with individual modules distinguished by unique colors. The most significantly associated module was selected for downstream analysis.

### 2.3 PPI

To investigate protein–protein interaction (PPI) relationships between PE-associated secreted proteins and key genes implicated in DD, a PPI network was constructed via the STRING database (version 12.0, https://www.string-db.org), with an interaction confidence score threshold set at 0.4 (28). The resulting network was visualized via Cytoscape software (version 3.8.2), and functional module identification was performed via the MCODE plugin. The MCODE parameters were configured as follows: degree cut-off = 2, node score cut-off = 0.2, k-core = 2, and maximum depth = 100. The three functional modules with the highest scores were selected for downstream bioinformatic analyses.

### 2.4 Functional enrichment analysis

To elucidate the functional characteristics and biological pathways associated with candidate genes related to PE and DD, Gene Ontology (GO) and Kyoto Encyclopedia of Genes and Genomes (KEGG) enrichment analyses were performed via the “clusterProfiler” and “enrichplot” packages in R. Gene annotation data were obtained from the “org.Hs.eg.db” file available in the Bioconductor database. The enrichment results were visualized via the “ggplot2” package.

### 2.5 Machine learning

To identify potential diagnostic biomarkers for DD associated with PE, two machine learning approaches were employed for gene selection. Initially, feature selection was performed via least absolute shrinkage and selection operator (LASSO) logistic regression implemented via the “glmnet” package in R (29). The gene importance was subsequently evaluated via the random forest (RF) algorithm via the “randomForest” package (30). Genes identified by both LASSO regression and RF analysis were intersected and considered candidate diagnostic markers for PE-associated DD.

### 2.6 Nomogram construction and model evaluation

To assess the diagnostic efficacy of candidate genes for PE-related DD, a multivariate logistic regression model was established by integrating key hub genes via the “Irm” package in R to predict the risk of disease. A nomogram was generated to intuitively display the model and variable contributions (31). The model’s discrimination ability was evaluated via receiver operating characteristic (ROC) curves, and the area under the curve (AUC) was calculated. Calibration curves were used to assess the consistency between the predicted and observed probabilities. Decision curve analysis (DCA) was further applied to quantify the clinical benefit of the model. External validation of the nomogram’s predictive accuracy was performed using the independent DD cohort GSE98793.

### 2.7 Immune infiltration analysis and GSEA

Immune cells exhibit distinct patterns of infiltration and residency during disease initiation and progression, offering crucial insights into their roles in pathophysiological mechanisms. On the basis of the GSE39653 dataset of DD, the CIBERSORT algorithm was employed to quantify the relative infiltration levels of immune cells within disease tissues. The Wilcoxon rank-sum test was used to assess the differences in the proportions of 22 immune cell types between the DD group and healthy controls.FDR correction was applied to adjust for multiple comparisons. Spearman’s rank correlation analysis was conducted to evaluate the relationship between the expression levels of diagnostic biomarkers and immune cell infiltration. Furthermore, gene set enrichment analysis (GSEA) of characteristic genes was performed via the “org.Hs.eg.db” and “clusterProfiler” packages. Significantly enriched signalling pathways in DD were visualized via the “enrichplot” package.

### 2.8 Regulatory network analysis of hub genes

Putative transcription factors (TFs) associated with the hub genes were predicted via the KnockTF v2.0 database (https://bio.liclab.net/KnockTFv2/index.php). To visualize the regulatory mechanisms of the identified hub genes, a TF–hub gene regulatory network was constructed via Cytoscape software.

### 2.9 Molecular docking

To investigate potential environmental exposures associated with the characteristic genes, chemical compounds linked to these genes were retrieved from the Comparative Toxicogenomics Database (CTD, https://ctdbase.org/), with a particular focus on phthalates. A gene–chemical interaction network was constructed via Cytoscape to delineate potential roles of the identified genes within toxicological networks. To further explore the molecular interaction mechanisms between phthalates and key hub gene products, molecular docking simulations were performed via AutoDock Vina, and three-dimensional visualization of docking conformations was conducted via PyMOL. Protein structural data for target genes were obtained from the Protein Data Bank (PDB), while ligand structures were retrieved from the PubChem database.

### 2.10 Statistical analysis and visualization

R software (version 4.4.1) and various packages were used for statistical analysis and visualization. A p value of less than 0.05 was considered statistically significant.

## 3 Results

### 3.1 Differential expression gene screening in PE

**Figure 1:**
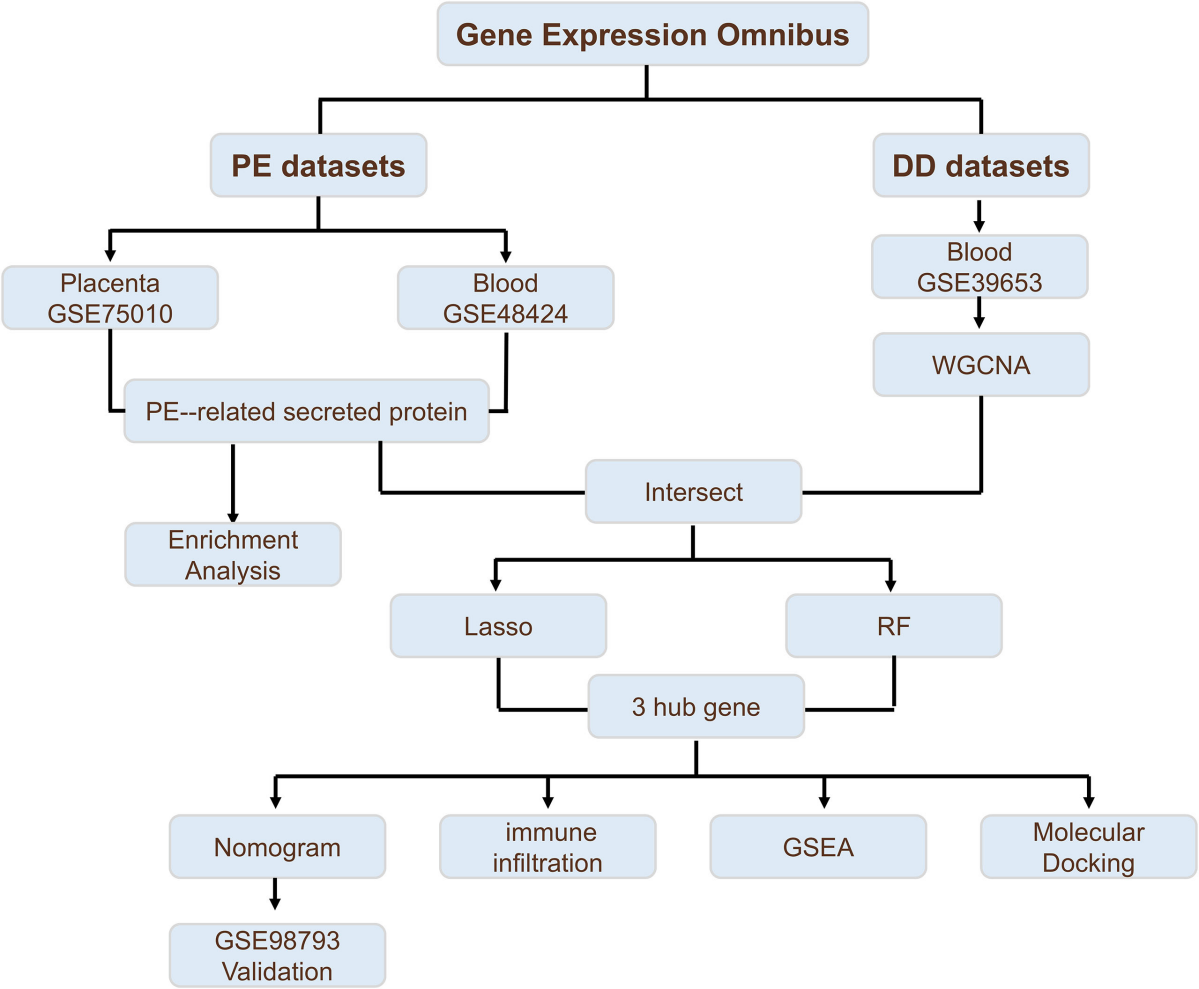
Integrated workflow of this study.

**Figure 2:**
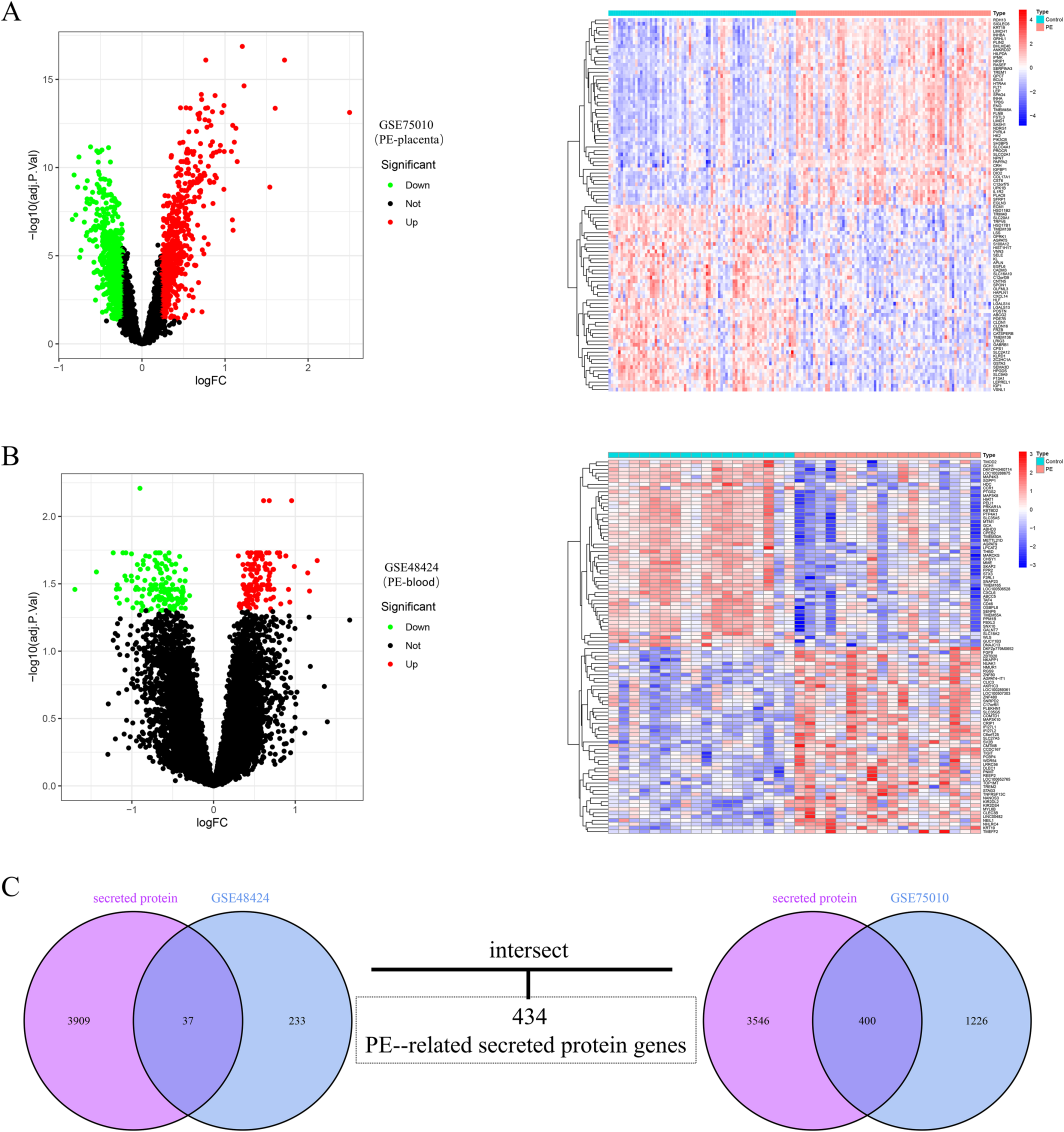
Integration and differential expression analysis of placental and blood datasets from preeclampsia patients. **(A)** Volcano plot of DEGs in placental tissues from preeclampsia patients, alongside a heatmap of the top 50 upregulated and downregulated DEGs. **(B)** Volcano plot of DEGs in peripheral blood samples from preeclampsia patients, accompanied by a heatmap displaying the top 50 upregulated and downregulated DEGs. The upregulated genes are indicated by red dots, and the downregulated genes are indicated by green dots. **(C)** Venn diagram showing the overlap between placental tissue and peripheral blood DEGs and secretory protein-coding genes, identifying 434 preeclampsia-related secretory protein genes.

The analytical workflow of this study is illustrated in **Figure 1**. Two raw datasets, comprising samples from PE patients and healthy controls, were downloaded from the GEO database. After normalization of the raw microarray data, differential expression analysis was performed via the “limma” package. The results revealed a total of 1,626 differentially expressed genes (DEGs) in placental tissue, including 781 upregulated and 845 downregulated genes. In peripheral blood samples, 270 DEGs were detected, comprising 130 upregulated and 140 downregulated genes. The expression patterns of DEGs in placental tissue and peripheral blood from PE patients are visually presented via volcano plots and heatmaps (**Figures 2A, B**). Furthermore, to investigate PE-associated secretory proteins, a set of 3,946 human genes encoding secreted proteins was intersected with the PE DEGs. This analysis revealed 434 secretory protein genes related to PE, including 400 derived from placental tissue and 37 from peripheral blood samples (**Figure 2C**).

### 3.2 Coexpression network construction and core module identification

**Figure 3:**
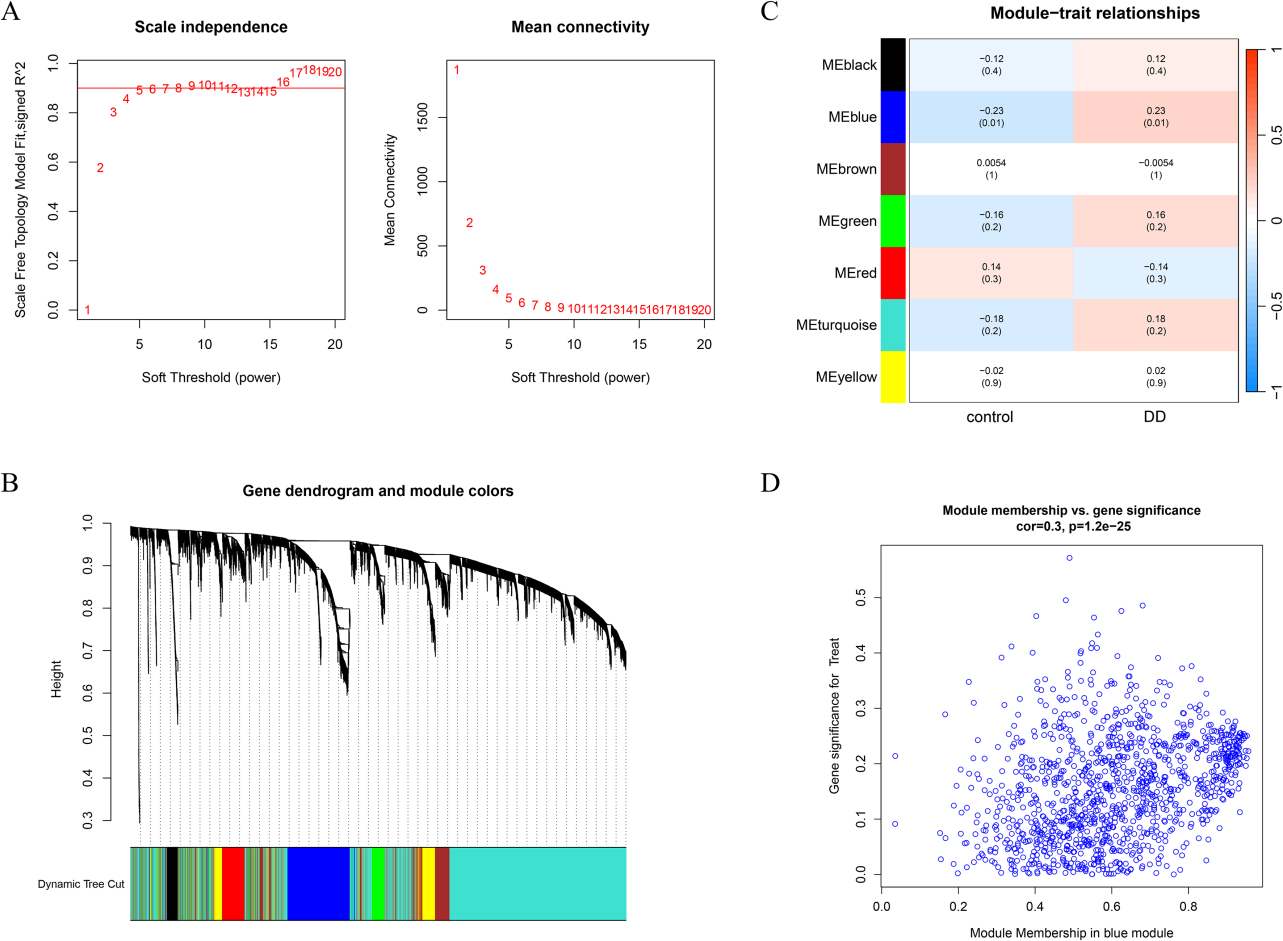
Identification of disease-associated gene modules via WGCNA. **(A)** Determination of the optimal soft-thresholding power β on the basis of the scale-free topology model. A soft threshold of β = 4 was selected according to mean connectivity and scale independence. **(B)** Gene clustering dendrogram for DDs with different colors representing distinct gene modules. **(C)** Heatmap depicting correlations between module eigengenes and disease phenotypes in DD. Blue indicates a negative correlation, and red indicates a positive correlation. **(D)** Scatter plot illustrating the correlation between gene significance and module membership in the blue module.

Weighted gene coexpression network analysis (WGCNA) was performed on the depression dataset GSE39653 to identify the gene modules most closely associated with depression. A soft-thresholding power of β=10 was chosen to ensure that the constructed coexpression network conformed to scale-free topology and met the average connectivity criteria (**Figure 3A**). The minimum module size was set to 150, and a module merging threshold of 0.25 was applied. Hierarchical clustering dendrograms for depression-related modules were generated (**Figure 3B**). Seven coexpression modules, each assigned a distinct color, were identified. Correlation analysis revealed that the blue module presented the strongest positive correlation with DD (r = 0.23, p = 0.01) (**Figure 3C**). Additionally, 19 gene modules were identified within the depression dataset, among which the MEblack module showed the strongest negative correlation (**Figure 3D**). Consequently, 1,165 key genes from the gray module were selected for subsequent analysis (**Figure 3D**).

### 3.3 Protein–protein interaction network and functional enrichment analysis of PE-associated secreted proteins and DD-related pathogenic genes

**Figure 4:**
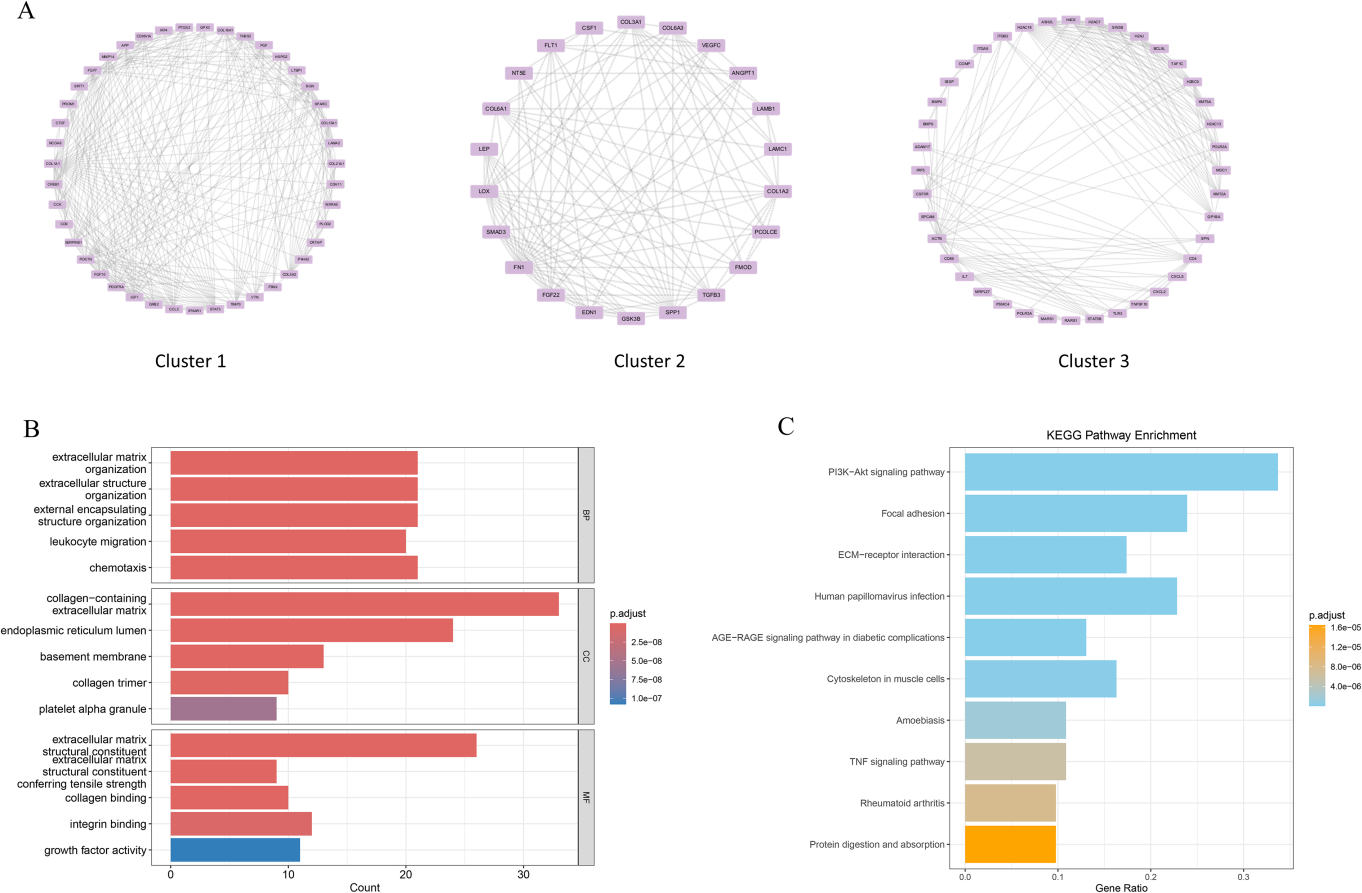
PPI analysis of PE-related secretory proteins and key DD genes. **(A)** Construction of the PPI network on the basis of high-scoring module genes identified via the MCODE plugin in Cytoscape. **(B)** Gene Ontology (GO) enrichment analysis results are presented as bar plots. **(C)** Kyoto Encyclopedia of Genes and Genomes (KEGG) pathway enrichment analysis results are shown as bar plots.

Clinical studies have revealed that patients with preeclampsia (PE) have a greater risk of developing depressive disorder (DD), suggesting a potential causal link between these two conditions. To investigate this association, a protein–protein interaction (PPI) network comprising 434 PE-associated secreted proteins and 1,165 MDD-related pathogenic genes was constructed via the STRING database. The PPI network was further analysed via the MCODE plugin in Cytoscape, which identified 24 significant modules. Among these, the top three modules based on MCODE scores contained a total of 105 genes, which were considered candidate DD pathogenic genes associated with PE (**Figure 4A**). GO enrichment analysis indicated that these genes were predominantly involved in biological processes such as leukocyte migration, cell chemotaxis, and positive regulation of cytokine production. These genes were localized to cellular components, including the collagen-containing extracellular matrix and the endoplasmic reticulum lumen, and were functionally associated with cytokine activity and chemokine activity (**Figure 4B**). KEGG pathway analysis revealed that these genes were significantly enriched in the PI3K-Akt signalling pathway, focal adhesion, and ECM-receptor interaction. Moreover, these genes were implicated in disease-associated pathways, including human papillomavirus infection, AGE-RAGE signalling in diabetic complications, and rheumatoid arthritis (**Figure 4C**). Collectively, these findings suggest that the identified genes may play critical roles in immune regulation, cell migration, and inflammatory responses.

### 3.4 Identification of diagnostic biomarkers for PE-associated DD via machine learning

**Figure 5:**
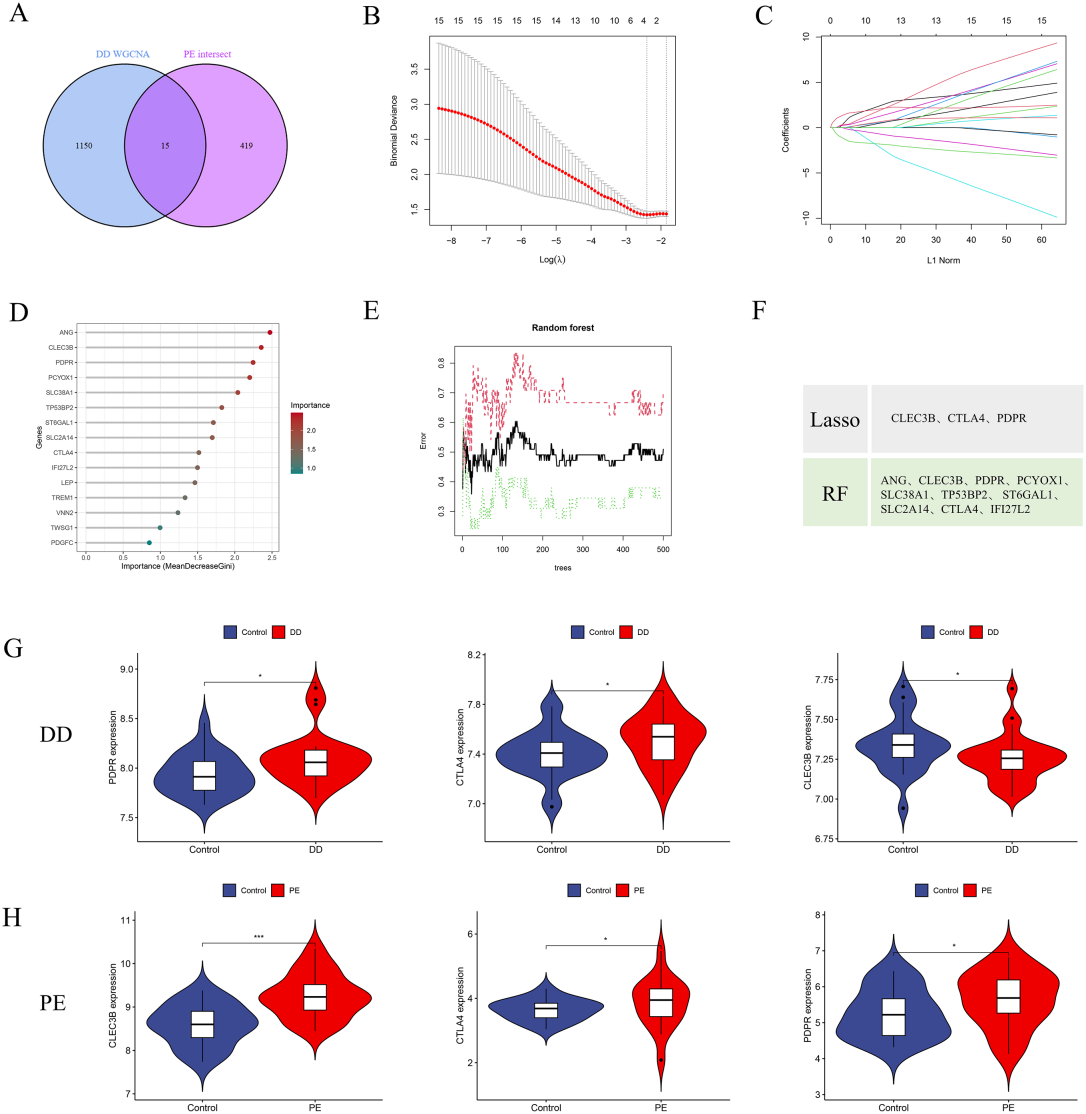
Identification of potential diagnostic biomarkers for PE-associated DD via machine learning approaches. **(A)** Venn diagram illustrating 15 intersecting genes between PE-related secretory proteins and key depressive disorder (DD) pathogenic genes. **(B, C)** Selection of the optimal minimum error and corresponding lambda value for biomarker screening on the basis of the LASSO logistic regression algorithm. **(D, E)** Bar plots displaying the importance rankings of 21 candidate genes according to MeanDecreaseGini derived from the RF algorithm. **(F)** Intersection of genes selected by the LASSO and RF algorithms, resulting in three potential pathogenic signature genes (CLEC3B, CTLA4, and PDPR). **(G, H)** Comparative expression analysis of the three signature genes in the depressive disorder dataset (GSE39653) and the PE dataset. *p < 0.05; **p < 0.01, ***p < 0.001.

PE-associated secreted proteins have been implicated in the pathogenesis and progression of DD. To identify potential biomarkers, an integrative analysis combining PE-associated secreted proteins with major depressive disorder (MDD)-related pathogenic genes was conducted, resulting in the identification of 15 candidate genes (**Figure 5A**). On the basis of these candidates, a diagnostic model was constructed to identify patients with comorbid PE and DD. LASSO regression was subsequently applied to refine the 15-gene set, yielding three potential pathogenic genes: CLEC3B, CTLA4, and PDPR (**Figures 5B, C**). In parallel, the RF machine learning algorithm was employed to rank candidate genes according to the mean decrease in the Gini index, and genes with MeanDecreaseGini ≥ 1.5 were selected (**Figures 5D, E**). An intersection analysis of the LASSO and RF results revealed three overlapping feature genes (**Figure 5F**).

Gene expression analysis of the GSE39653 dataset revealed significantly elevated levels of CLEC3B, CTLA4, and PDPR in DD patients than in healthy controls (**Figure 5G**) (P < 0.05). Similarly, in PE patients, the expression of these genes was also significantly upregulated relative to that in normal subjects (**Figure 5H**) (P < 0.05). These findings suggest that CLEC3B, CTLA4, and PDPR are consistently upregulated in both DD and PE patients and may serve as diagnostic biomarkers for identifying individuals with concurrent inflammatory or vascular dysfunction associated with depressive pathology.

### 3.5 Construction of a predictive nomogram model for PE-associated DD

**Figure 6:**
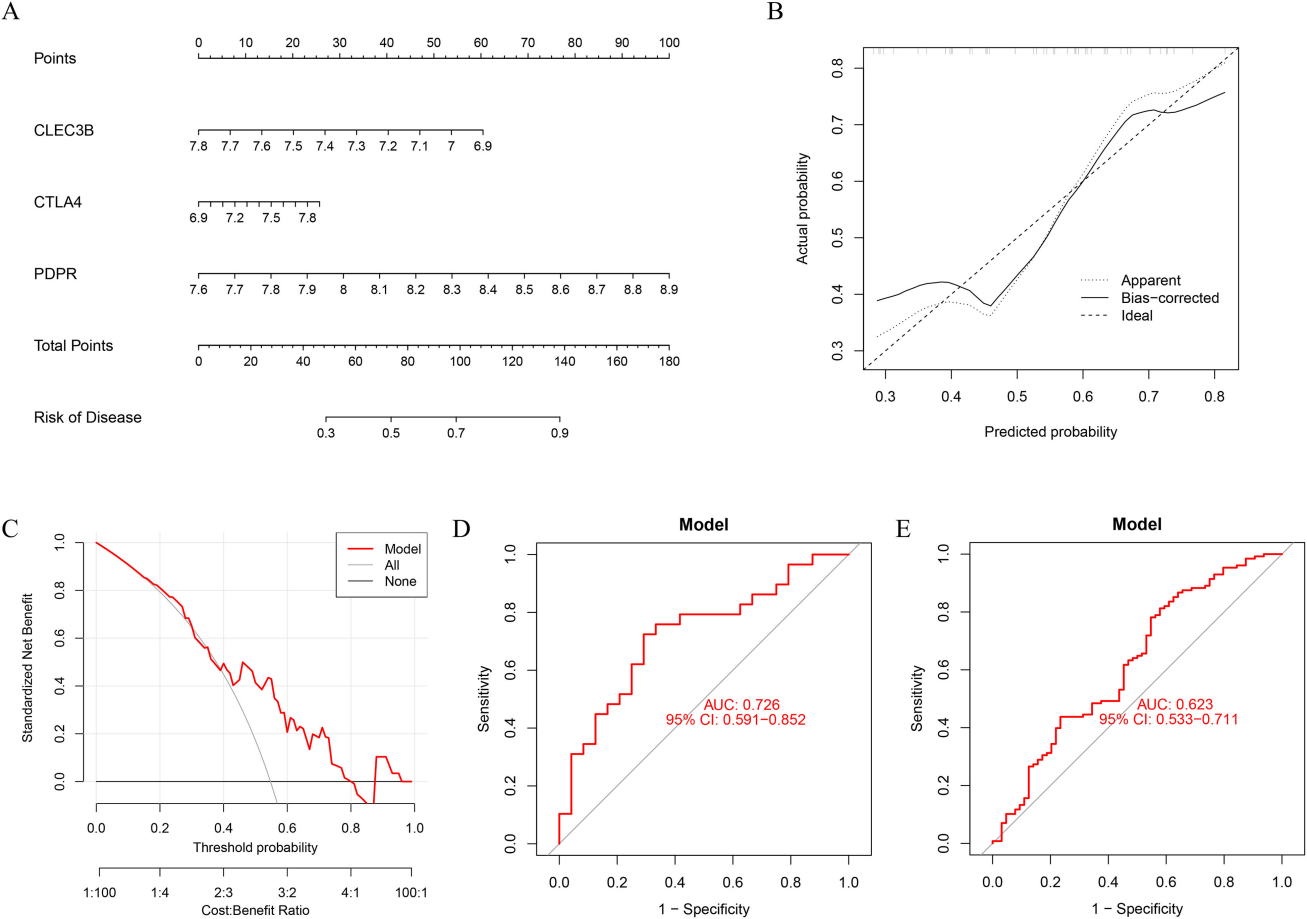
Construction and performance evaluation of the diagnostic nomogram model. **(A)** A nomogram prediction model was constructed based on the selected diagnostic biomarkers. **(B)** Calibration curve of the nomogram showing the agreement between the predicted probabilities and the actual incidence of PE-related depressive disorder. **(C)** Decision curve analysis indicating the clinical net benefit of the nomogram model in comparison with the assumption that all or none of the patients had depressive disorders. The black line represents the net benefit when no patients are affected, the gray line represents all patients affected, and the red line represents the model’s predicted net benefit. **(D)** The nomogram achieved an AUC of 0.726 in the internal validation dataset GSE39653, indicating good diagnostic performance. **(E)** The ROC curve in the external dataset GSE98793 had an AUC of 0.623, supporting its moderate generalization ability.

To improve the diagnostic and predictive accuracy of DD associated with PE, a nomogram prediction model was developed on the basis of three selected feature genes via logistic regression analysis (**Figure 6A**). The cumulative scores corresponding to each feature gene were summed to calculate a total score, which was subsequently used to estimate the risk of PE-associated DD onset. Calibration curve analysis demonstrated high concordance between the predicted probabilities of the nomogram model and the ideal reference model (**Figure 6B**). Furthermore, decision curve analysis (DCA) was employed to evaluate the clinical utility of the model. The results indicated that the intervention strategy guided by the model yielded greater net benefit than did the intervention strategy in all patients or no intervention across most threshold probabilities (**Figure 6C**). The diagnostic sensitivity and specificity of the nomogram model for PE-associated DD were assessed via receiver operating characteristic (ROC) curve analysis, with an area under the curve (AUC) of 0.726 in the internal validation dataset GSE39653 (**Figure 6D**). In the external validation dataset GSE98793, the model achieved an AUC of 0.623 for diagnosing DD patients (**Figure 6E**), suggesting satisfactory predictive performance for depressive disorders related to inflammatory bowel disease.

### 3.6 GSEA-based functional enrichment and immune infiltration analysis

**Figure 7:**
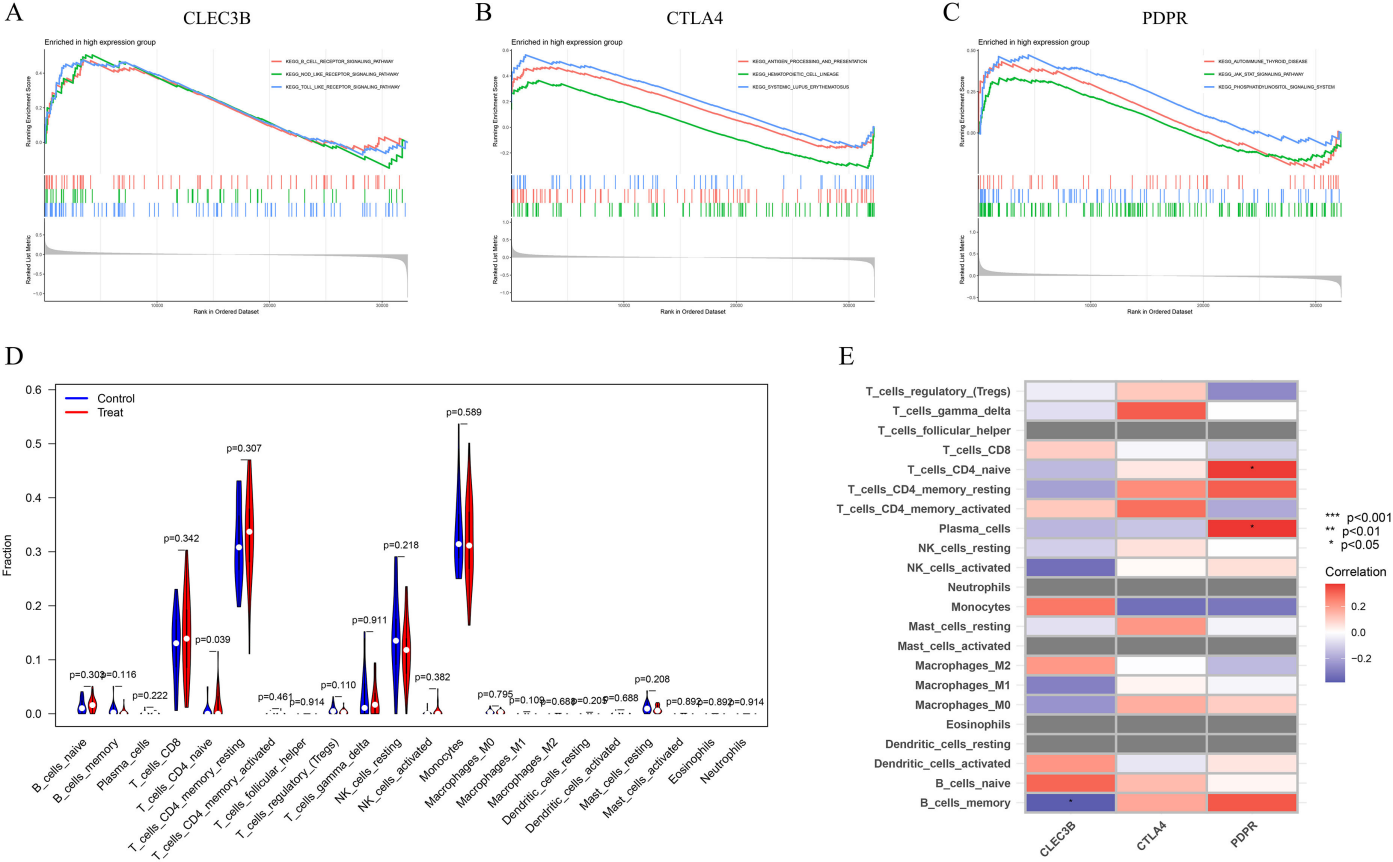
GSEA and immune cell infiltration analysis of candidate biomarkers. **(A)** GSEA and KEGG pathway enrichment were performed for the three characteristic genes to explore their potential roles in immune regulation and related signalling pathways. **(B)** Violin plot illustrating the differences in the infiltration levels of 22 immune cell subtypes between the DD group and healthy controls. **(C)** Heatmap showing the correlations between the expression of characteristic genes and the infiltration levels of immune cells.

GSEA was conducted to investigate the potential biological functions of three feature genes within the DD dataset GSE39653. The analysis revealed that the enriched KEGG pathways among the highly expressed feature genes predominantly involved the B-cell receptor, Toll-like receptor, and NOD-like receptor signalling pathways, along with antigen processing and presentation. The activation of the JAK-STAT and phosphatidylinositol signalling pathways further supported an immune-activated status, suggesting that these three genes may mediate immune regulation in DD (**Figures 7A–C**). The CIBERSORT algorithm was subsequently applied to estimate the infiltration levels of 22 immune cell types in DD samples. The results revealed a significant increase in naive CD4+ T cells (T_cells_CD4_naive) in the DD group (**Figure 7D**). Correlation analyses further demonstrated a negative association between CLEC3B and memory B cells (B_cells_memory), whereas PDPR was positively correlated with naive CD4+ T cells and plasma cells.

### 3.7 Construction of the feature gene–transcription factor network and molecular docking analysis with plasticizers

**Figure 8:**
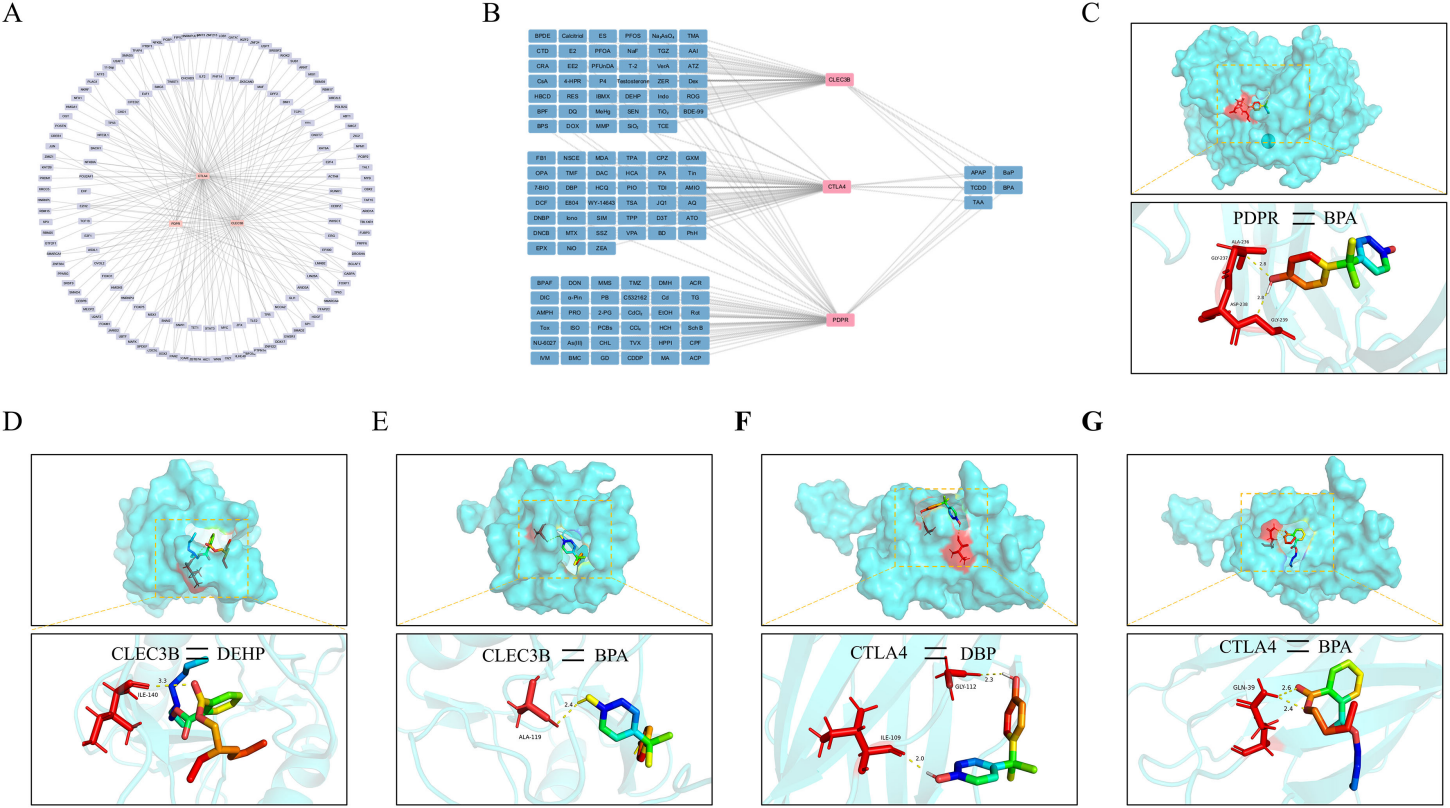
Transcriptional regulatory network construction and molecular docking analysis **(A)** Upstream transcription factors of the characteristic genes were predicted to construct a regulatory network comprising transcription factors and their target genes to uncover potential regulatory mechanisms. **(B)** A network was established to illustrate the interactions between characteristic genes and small-molecule compounds, identifying potential phthalates. **(C)** Molecular docking simulation demonstrating the binding of the PDPR protein with BPA. **(D, E)** Predicted docking structures of the CLEC3B protein with DEHP and BPA. **(F, G)** Docking models of the CTLA4 protein with DBP and BPA were simulated.

To elucidate the regulatory landscape of the three feature genes and their interacting components, a comprehensive gene–transcription factor (TF) regulatory network was constructed (**Figure 8A**). These feature genes are regulated by multiple transcription factors, suggesting that their functions in disease pathogenesis are tightly controlled by complex transcriptional programs. Accumulating evidence highlights the immunotoxic potential of plasticizers, underscoring their relevance to human immune modulation. To explore potential binding interactions between plasticizers and key genes associated with PE-related DD, a gene–compound interaction network was constructed via the Comparative Toxicogenomics Database (CTD) (**Figure 8B**). PDPR exhibited a stable binding affinity with bisphenol A (BPA), primarily forming hydrogen bonds at the ALA-3236 and GLY-239 residues (**Figure 8C**). CLEC3B interacted with both BPA and DEHP, with molecular docking analysis revealing minimum binding energies of –6.4 kcal/mol and –5.4 kcal/mol, respectively (**Figures 8D, E**). CTLA4 was found to interact with BPA and DBP. BPA formed hydrogen bonds at GLY-112 and ILE-109, whereas DBP bound at GLN-39 (**Figure 8F, G**). These findings indicate that these feature genes exhibit broad binding potential to various plasticizer components, indicating their toxicological involvement in the pathogenesis of PE-associated DD.

## 4 Discussion

Accumulating evidence indicates a close association between preeclampsia (PE) and depression (32). As a common complication during pregnancy, PE has lasting impacts on both maternal and fetal health and may significantly affect postpartum psychological well-being (33). Clinically, depression is frequently diagnosed among PE patients, although the underlying mechanisms remain unclear. Studies have demonstrated that, compared with normal pregnancies, PE markedly increases the risk of postpartum depression (34). Buglione-Corbett et al. identified overlapping inflammatory biomarkers between PE and depression, suggesting shared molecular pathways (35). Elucidating these molecular mechanisms is crucial for the early identification and intervention of high-risk populations.

By integrating gene expression profiles from PE and DD cohorts, this study applied diverse bioinformatics methods to pinpoint 434 secretory protein genes linked to PE and 1,165 pathogenic genes associated with depression. Analysis of the PPI network revealed 105 pivotal candidate genes, suggesting functional module-based crosstalk between these diseases. GO and KEGG pathway enrichment revealed that these overlapping genes are involved primarily in immune modulation and cell signalling pathways, including transforming growth factor beta (TGF-β) biosynthesis, leukocyte activation, and tumor necrosis factor (TNF) signalling. The enrichment of the PI3K-Akt signalling cascade, extracellular matrix (ECM)-receptor interactions, and AGE-RAGE pathways implicated in diabetic complications highlights their key roles in inflammatory responses and cellular migration, reinforcing the hypothesis that PE and depression are linked through shared inflammatory mechanisms. These results point to the molecular connections between PE and depression driven by immune system dysregulation and disrupted signalling pathways, offering new perspectives on their comorbidity. Research has indicated that immune dysfunction in PE contributes to placental abnormalities and systemic inflammation (36), whereas chronic inflammation is a recognized contributor to depression pathogenesis (37). The identification of intersecting pathways, such as the TNF signalling pathway, corroborates these findings (38). This study’s cross-disease integrative strategy reveals disease-specific molecular mechanisms connecting PE and depression via immune and signalling disturbances, expanding the understanding of their comorbid characteristics and establishing a foundation for subsequent investigations.

High-throughput sequencing advancements have enabled comprehensive investigations into disease comorbidity mechanisms. WGCNA was performed on the depression dataset GSE39653 to construct coexpression networks and identify modules and hub genes closely linked to DD. A total of 19 modules were detected, with the blue module showing a significant positive association with DD. From the gray module, 1,165 key genes were extracted for further integrative analyses, highlighting their potential central roles in depression pathophysiology and indicating that specific module genes may have pathogenic functions under disease conditions. The construction of accurate diagnostic models alongside detailed immune characterization remains essential for deciphering complex disease mechanisms (39, 40). Using LASSO regression and random forest (RF) methods, CLEC3B, CTLA4, and PDPR were identified as key feature genes. CLEC3B, a member of the C-type lectin domain family, is involved in extracellular matrix remodelling and immune response regulation and serves as a biomarker in several inflammatory disorders (41, 42). CTLA4 is a well-known immune checkpoint that modulates immune tolerance and autoimmunity by suppressing T-cell activation (43). PDPR, a regulatory subunit of pyruvate dehydrogenase phosphatase, regulates mitochondrial metabolism, energy homeostasis, and oxidative stress (44), potentially impacting neuronal metabolic stability. The resulting diagnostic model achieved AUCs of 0.726 and 0.623 in the internal and external validation cohorts, respectively, demonstrating reliable predictive performance. Moreover, gene set enrichment analysis (GSEA) suggested that these genes may contribute to immune activation via the Toll-like receptor, NOD-like receptor, and JAK-STAT pathways, further corroborating their involvement in inflammatory regulation.

Immune cell infiltration in DD samples was estimated via the CIBERSORT algorithm, which revealed a significant increase in naive CD4+ T cells in patients with DD. Correlation analysis further demonstrated a significant positive association between PDPR and naive CD4+ T cells, whereas CLEC3B was negatively correlated with memory B cells. These findings suggest that these three candidate genes may regulate inflammation by modulating the infiltration of specific immune cell subsets, thereby mediating the comorbidity between PE and DD. It has been reported that the activation and migration of immune cells, including CD4+ T cells, B cells, and regulatory T cells, in PE patients contribute to endothelial dysfunction and placental inflammation, promoting pathological progression (4, 45). Additionally, these immune cells participate in immune–inflammatory regulatory networks through the secretion of inflammatory cytokines and the activation of signalling pathways such as the JAK-STAT and Toll-like receptor pathways (46, 47). On the basis of immune cell correlations and the constructed gene–transcription factor (TF) regulatory network, this study elucidates the pivotal roles of CLEC3B, CTLA4, and PDPR within a complex regulatory framework. These genes likely influence immune cell functional states and inflammatory responses through multilayered transcriptional regulation, thereby facilitating the pathological intersection and comorbidity of PE and depression.

Phthalates, which are widely recognized as prevalent environmental immunotoxicants, have been implicated in disrupting immune homeostasis amid rising global pollution concerns (48, 49). Despite growing knowledge about their immunological impacts, the disease-specific consequences of phthalate exposure—particularly in conditions such as preeclampsia (PE) and depression—remain underexplored. Here, molecular docking analyses revealed that CLEC3B, CTLA4, and PDPR exhibit stable binding affinities with common phthalates such as BPA, DEHP, and DBP. PDPR, in particular, strongly bound with BPA at ALA-3236 and GLY-239, with a minimum binding energy of –6.4 kcal/mol, suggesting a possible role in immunotoxic processes. These results align with previous findings on phthalate-mediated immune regulation and support the notion that environmental toxins can exacerbate disease through immune perturbation (50). Importantly, this study shifts the focus from broad toxicological profiles to pinpointing specific molecular targets, identifying three candidate genes as potential mechanistic bridges between environmental exposure and PE–depression comorbidity, with notable implications for targeted intervention and biomarker development.

In addition to these findings, it is essential to acknowledge the limitations inherent in the present study, particularly the lack of detailed clinical information in the utilized datasets, such as phthalate exposure levels, depression severity scores, gestational age, and treatment history. Additionally, it should be noted that the depression datasets analyzed in this study encompass DD in a broad sense and are not specific to perinatal or postnatal depression, which may exhibit distinct biological underpinnings. This difference could affect the generalizability of the identified blood-derived signatures to the pregnancy context. Future studies should integrate multi-center prospective clinical data with well-characterized patient phenotypes and employ *in vivo* and *in vitro* validation to substantiate the mechanistic roles of the identified biomarkers. Such efforts will be crucial for translating these bioinformatics findings into clinically actionable tools.

In summary, this study reveals potential shared mechanisms between PE and DD involving immune dysregulation and aberrant cellular signal transduction. An integrated analysis identified 434 preeclampsia-associated secretory protein genes and 1165 depression-related pathogenic genes, from which three signature biomarkers, CLEC3B, CTLA4, and PDPR, were ultimately determined via LASSO regression and random forest algorithms. These genes were significantly upregulated under both conditions, and GSEA along with immune infiltration analyses supported their involvement in inflammatory and immune regulation through classical pathways such as Toll-like receptors, NOD-like receptors, and the JAK–STAT signalling axis. Further molecular docking analysis demonstrated stable binding interactions between these gene products and common plasticizers, including BPA, DEHP, and DBP, suggesting their potential role as molecular intermediaries in environmental toxin-induced immune responses. This work not only advances the understanding of the comorbidity between preeclampsia and depression but also offers novel biomarkers and research directions for early diagnosis and precision intervention.

## Data Availability

The original contributions presented in the study are included in the article/**Supplementary Material**. Further inquiries can be directed to the corresponding authors.
